# Propensity score‐matched comparison of stenting as a bridge to surgery and emergency surgery for acute malignant left‐sided colonic obstruction

**DOI:** 10.1186/s12893-021-01144-z

**Published:** 2021-03-20

**Authors:** Yuepeng Cao, Qing Chen, Zhizhan Ni, Feng Wu, Chenshen Huang, Jinzhe Zhou, Songze Zhang, Bujun Ge, Qi Huang

**Affiliations:** 1 Department of General Surgery, Tongji Hospital, Tongji University School of Medicine, 389 Xincun Road, 200065 Shanghai, China; 2 Department of Colorectal Surgery, Ningbo First Hospital, 315010 Ningbo, China; 3 Department of General Surgery, Shanghai Tenth People’s Hospital, Tongji University School of Medicine, 200072 Shanghai, China; 4 Department of General Surgery, Hwa Mei Hospital, University of Chinese Academy of Sciences, 315010 Ningbo, China

**Keywords:** Acute malignant left‐sided colonic obstruction, Self‐expanding metallic stent, Propensity score matching, Overall survival

## Abstract

**Background:**

Bridge to elective surgery (BTS) using self-expanding metal stents (SEMSs) is a common alternative to emergency surgery (ES) for acute malignant left-sided colonic obstruction (AMLCO). However, studies regarding the long-term impact of BTS are limited and have reported unclear results.

**Methods:**

A multicenter observational study was performed at three hospitals from April 2012 to December 2019. Propensity score matching (PSM) was introduced to minimize selection bias. The primary endpoint was overall survival. The secondary endpoints included surgical approaches, primary resection types, total stent-related adverse effects (AEs), surgical AEs, length of hospital stay, 30-day mortality and tumor recurrence.

**Results:**

Forty-nine patients in both the BTS and ES groups were matched. Patients in the BTS group more often underwent laparoscopic resection [31 (63.3%) vs. 8 (16.3%), p < 0.001], were less likely to have a primary stoma [13 (26.5%) vs. 26 (53.1%), p = 0.007] and more often had perineural invasion [25 (51.0 %) vs. 13 (26.5 %), p = 0.013]. The median overall survival was significantly lower in patients with stent insertion (41 vs. 65 months, p = 0.041). The 3-year overall survival (53.0 vs. 77.2%, p = 0.039) and 5-year overall survival (30.6 vs. 55.0%, p = 0.025) were significantly less favorable in the BTS group. In multivariate Cox regression analysis, stenting (hazard ratio(HR) = 2.309(1.052–5.066), p = 0.037), surgical AEs (HR = 1.394 (1.053–1.845), p = 0.020) and pTNM stage (HR = 1.706 (1.116–2.607), p = 0.014) were positively correlated with overall survival in matched patients.

**Conclusions:**

Self-expanding metal stents as “a bridge to surgery” are associated with more perineural invasion, a higher recurrence rate and worse overall survival in patients with acute malignant left-sided colonic obstruction compared with emergency surgery.

## Background

Colorectal cancer (CRC) is the third most commonly diagnosed cancer and accounts for approximately 10 % of cancer-related deaths worldwide [1]. A total of 7–29 % of CRC patients present with acute large bowel obstruction [2]. These patients are mainly in advanced disease stages and exhibit poor clinical conditions, and most of them need urgent surgical interventions [3]. In addition to the high morbidity and mortality rates of acute malignant colonic obstruction (AMCO) itself in the emergency setting, conventional emergency surgery (ES) is frequently followed by severe complications, such as anastomotic leakage and surgical site infection [4, 5]. Moreover, ES is accompanied by a high risk of stoma creation, which often becomes permanent [6].

In the 1900 s, self-expanding metal stents (SEMSs) were first utilized in the treatment of palliative malignant rectal obstruction [7]. Since then, SEMSs have been increasingly used as an alternative option for AMCO treatment either as palliation or as a bridge to surgery (BTS). BTS provides a period of “optimization” of the patients’ clinical condition, allowing adequate oncological staging, accurate anesthetic assessment, optimal colonic preparation and early peri-operational chemotherapy [2, 5]. Therefore, decompression by SEMS insertion can transform the ES into an elective procedure, leading to subsequent medical stabilization and an increased rate of primary anastomosis. SEMS seems to be an effective and safe technique, and the benefits of short-term outcomes have been confirmed by recent meta-analyses compared to ES [8–10]. However, SEMS as BTS is associated with annoying stent-related complications, including perforation, stent migration and reobstruction [6, 11]. In addition, BTS has the potential risk of tumor cell dissemination after stent insertion, leading to worse oncologic outcomes [12, 13]. Moreover, randomized controlled trials (RCTs) and related meta-analyses comparing BTS and ES are limited to specific populations and provide inconsistent results on surgical and oncological outcomes. Most studies evaluating the outcomes of BTS are conducted at single centers, and optimal matching is often lacking [2, 5, 14]. The objective of this study was to compare the surgical and oncological outcomes of BTS and ES in the management of AMLCO using propensity score-matched (PSM) analysis.

## Methods

We performed a retrospective observational study of patients with AMLCO treated with curative intent, in three hospitals (Shanghai Tongji Hospital, Shanghai, China; Ningbo First Hospital, Ningbo, China; Ningbo Second Hospital, Ningbo, China) from April 2012 to December 2019. All patients who underwent resection for AMLCO were identified from in-hospital medical records. Data on baseline characteristics and short-term surgical outcomes were collected from medical records. Data on long-term oncological outcomes were collected upon follow-up treatment or survey. This study was approved by the institutional review board of Shanghai Tongji Hospital, Ningbo First Hospital and Ningbo Second Hospital.

### Patient selection

Patients were considered to have acute colonic obstruction based on clinical signs of colonic obstruction (abdominal distention, constipation and vomiting) and related radiological signs under computed tomography (CT) scan. Patients were then separated into 2 distinct groups based on the type of procedure performed. The BTS group included patients who underwent SEMS placement followed by scheduled elective surgery, and the ES group included patients who were treated with emergency surgery. Patients undergoing SEMS placement or ES as palliative treatment were excluded. Patients who were lost to follow-up were also excluded from the analysis. Patients who failed SEMS insertion procedure and underwent emergency surgery were classified in the BTS group according to the intention-to-treat concept.

The required sample size in each group was calculated using G*Power (Universitat Kiel, Germany) software. The primary endpoint of the present study was 5 year overall survival (OS). Previous studies reported that the 5-year OS after emergency surgery for acute malignant left-sided colonic obstruction was 40–80 % (1, 2). We assumed the 5 year OS to be 60 % based on previous studies and our clinical experience. However, compared to ES, the 5 year OS was difficult to calculate because studies on long-term outcomes were lacking. The difference in 5-year OS between BTS and OS was reported to be 42–60% (3, 4). Therefore, we assumed a 50% difference in 5-year OS for the 2 groups (BTS vs. ER 30 vs. 60%). A minimum sample size of 42 patients per group was estimated to obtain a power of 80% for detecting a difference at the 5% level. In our study, a total of 49 patients per group was included, with a G-power of 85.8%.

### Data recording and follow-up

Patient baseline characteristics (age, sex, ASA score, comorbidities, tumor location and clinical TNM stage) and perioperational characteristics (surgical approach, operation method, total hospital stay, stent-related adverse effects (AEs), surgical AEs and adjuvant chemotherapy) were collected from medical records. The metastasis found within 90 days after surgery was defined as pM1. Follow-up data, including tumor recurrence and overall survival, were obtained during routine clinical care and telephone contact with the patient.

### Stent placement and surgery

The colonic stents used in this study were uncovered and inserted with direct endoscopic visualization. All procedures were performed under general anesthesia. Stent placement was performed following standard protocols as previously described [15]. A guidewire was introduced across the stenosis and beyond the obstruction, and the stent was deployed over the guidewire. Correct positioning of the stent was confirmed by fluoroscopy.

Elective surgery was performed at a median of 11.5 days after stent insertion. All patients underwent standard colectomy and regional lymphadenectomy. The surgical approaches, operation methods and the range of resection were determined by the surgeon based on the tumor location, tumor stage and the patients’ general condition.

### Endpoints

The technical success of SEMS placement was defined by successful stent placement and the ability to pass stool. Clinical success was defined as relief of obstructive symptoms within 48 h of SEMS placement or ES. Stoma creation included temporary stoma with return after initial decompression surgery and permanent stoma according to the patient’s general condition, cancer progression and patient’s choice. The primary endpoint for this study was overall survival (OS). The secondary endpoints included surgical approach, primary resection type, total stent-related AEs (tumor perforation, bleeding and reobstruction), surgical AEs (wound infection, anastomosis leakage), length of hospital stay, 30-day mortality and tumor recurrence. Surgical AEs were classified according to the Clavien-Dindo classification [16].

### Statistical analysis

Propensity score matching (PSM) is used for best possible matching when randomized control trial (RCT) studies are not possible or not available. PSM represents the probability of receiving treatment A rather than B for a patient with given observed baseline characteristics with a summary score, the propensity score. The variables selected in PSM analysis included age, sex, ASA score, comorbidities, tumor location and clinical TNM (cTNM) stage. These variables were selected since they could affect the oncological outcomes of ACMO. PSM analysis was performed using a logistic regression model. This one-to-one matching was performed by using a caliper width that was 0.2 of the standard deviation of the log of the propensity score.

Data were analyzed using SPSS 22.0 (Chicago, IL, USA). Categorical variables were analyzed using the χ² test or Fisher’s exact test. Continuous data were analyzed using Student’ s t test and presented as the means ± standard deviation. Overall survival was defined as the time between diagnosis and the time of death or last follow-up. Survival curves were generated using the adjusted Kaplan-Meier method [17] and compared using a log-rank test. A p < 0.05 was considered statistically significant.

## Results

### Baseline characteristics

**Fig. 1:**
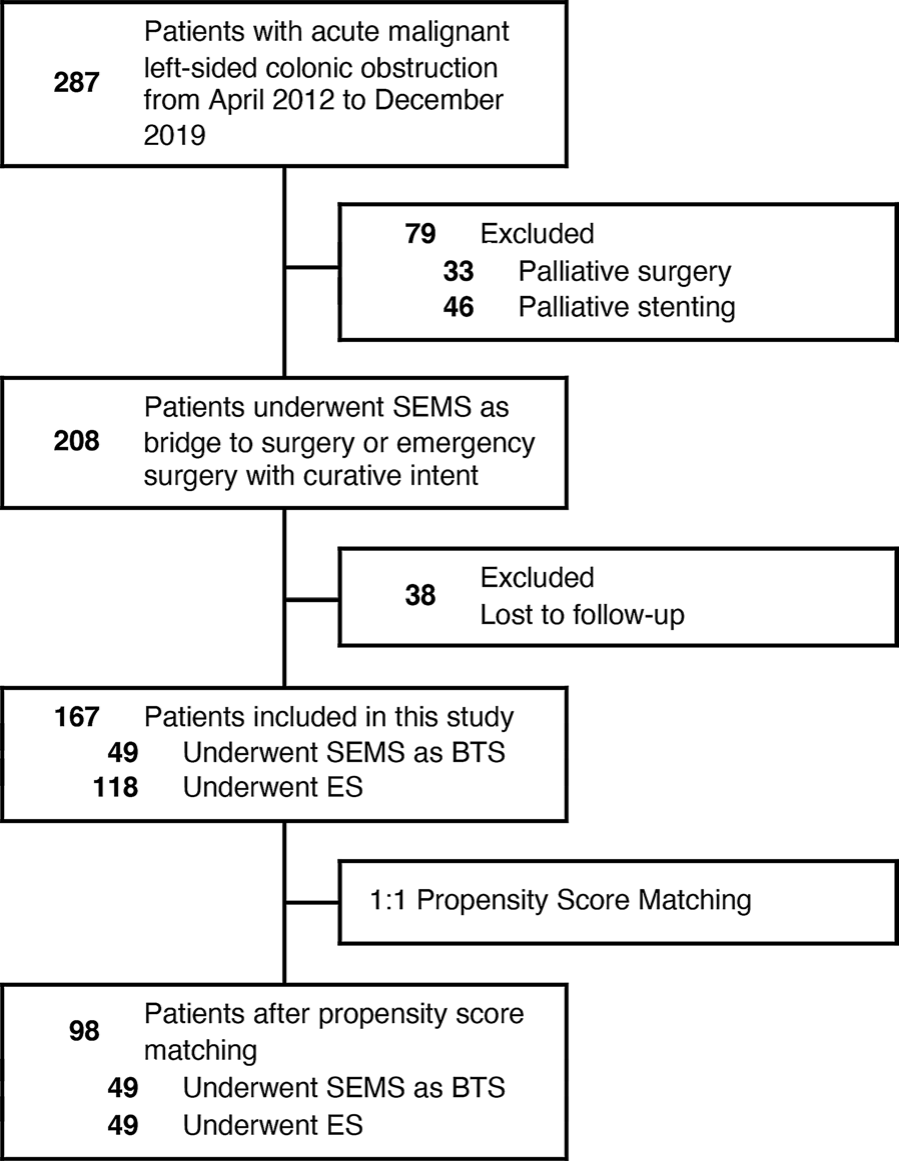
Flowchart of patient selection

From April 2012 to December 2019, we retrospectively identified 287 patients who were admitted for AMLCO in three hospitals. Patient selection is shown in Fig. 1. After exclusion of patients with palliative operation (n = 79) and patients with failed follow-up (n = 38), a total of 167 patients who received either SEMS as BTS (n = 49) or ES (n = 118) were included in the analysis. Using 1:1 propensity score matching, 49 patients in the ES group were matched to 49 in the SEMS group.

Before matching, baseline characteristics were comparable between the BTS and ES groups with the exception that more patients had coronary artery disease in the SEMS group (9 of 49 in the SEMS group (18.4 %) and 10 of 118 in the ES group (8.5 %)) (Table 1). After propensity matching, no significant difference was found between the BTS and ES groups regarding sex (36 male patients [73.5 %] in the BTS group and 37 [75.5 %] in the ES group, p = 0.817), age (mean [SD] age, 68.8 [11.1] years in the BTS group and 68.8 [13.8] years in the ES group, p = 0.872), age range (p = 0.757), ASA score (p = 0.399), comorbidities, tumor location (p = 0.243) or clinical TNM (cTNM) stage (2 patients (4.1 %) with stage IV CRCs in the BTS group and 1 (2.0 %) in the ES group, p = 0.560).

**Table 1:** Baseline characteristics

	Before propensity score matching			After propensity score matching(1:1)		
	BTS (n = 49)	ES (n = 118)	*P*	BTS (n = 49)	ES (n = 49)	*P*
Sex			0.152			0.817
Male	36 (73.5 %)	73 (61.9 %)		36 (73.5 %)	37 (75.5 %)	
Female	13 (26.5 %)	45 (38.1 %)		13 (26.5 %)	12 (24.5 %)	
Age(mean(SD),y)	68.8 (11.1)	68.3 (12.6)	0.811	68.8 (11.1)	68.8 (13.8)	0.872
Age range (y)			0.843			0.757
25–59	10 (20.4 %)	26 (22.0 %)		10 (20.4 %)	13 (26.5 %)	
60–69	18 (36.7 %)	36 (30.5 %)		18 (36.7 %)	15 (30.6 %)	
70–79	10 (20.4 %)	29 (24.6 %)		10 (20.4 %)	10 (20.4 %)	
80–95	11 (22.4 %)	27 (22.9 %)		11 (22.4 %)	11 (22.4 %)	
ASA score			0.348			0.399
I	6 (12.2 %)	28 (23.7 %)		6 (12.2 %)	13 (26.5 %)	
II	23 (46.9 %)	48 (40.7 %)		23 (46.9 %)	16 (32.7 %)	
III	17 (34.7 %)	40 (33.9 %)		17 (34.7 %)	18 (36.7 %)	
IV	3 (6.1 %)	2 (1.7 %)		3 (6.1 %)	2 (4.1 %)	
Comorbidities						
CAD	9 (18.4 %)	10 (8.5 %)	0.007	9 (18.4 %)	6 (12.2 %)	0.400
Hypertension	18 (36.7 %)	43 (36.4 %)	0.133	18 (36.7 %)	17 (34.7 %)	0.833
Diabetes	3 (6.1 %)	18 (15.3 %)	0.333	3 (6.1 %)	5 (10.2 %)	0.461
CPD	4 (8.2 %)	6 (5.1 %)	0.181	4 (8.2 %)	4 (8.2 %)	1.000
Renal dysfunction	2 (4.1 %)	5 (4.2 %)	0.705	2 (4.1 %)	3 (6.1 %)	0.646
Biliary diseases	6 (12.2 %)	14 (11.9 %)	0.412	6 (12.2 %)	5 (10.2 %)	0.749
CVD	2 (4.1 %)	6 (5.1 %)	0.873	2 (4.1 %)	3 (6.1 %)	0.646
Tumor location			0.086			0.243
Splenic flexure	8 (16.3 %)	10 (8.5 %)		8 (16.3 %)	5 (10.2 %)	
Descending colon	6 (12.2 %)	30 (25.4 %)		6 (12.2 %)	12 (24.5 %)	
Sigmoid colon	35 (71.4 %)	78 (66.1 %)		35 (71.4 %)	32 (65.3 %)	
cTNM stage			0.630			0.560
I–III	47 (95.9 %)	111 (94.1 %)		47 (95.9 %)	48 (98.0 %)	
IV	2 (4.1 %)	7 (5.9 %)		2 (4.1 %)	1 (2.0 %)	

*CAD* coronary artery disease, *CPD* chronic pulmonary disease, *CVD* cerebrovascular disease

Data are presented as the number (percentage) of patients unless otherwise indicated

### Procedure‐related characteristics and perioperational outcomes

SEMS insertion was attempted in 49 patients and was successful in 44 patients with a technical success rate of 89.8 %. The technique failure in 5 patients was attributed to a complete colonic obstruction, leading to unsuccessful stent insertions. Stent-related colonic perforation occurred in 2 of 44 patients within 24 h after stent insertion with a clinical success rate of 85.7 %. These 7 patients underwent emergency open surgery for tumor resection.

Table 2 summarizes the procedure-related characteristics and perioperational outcomes. The median interval between SEMS and resection was 11 days (IQR, 7–19 days). After PSM matching, patients in the BTS group more often underwent laparoscopic resection [31 (63.3%) in the BTS group and 8 (16.3%) in the ES group, p < 0.001], were less likely to have a primary stoma [13 (26.5%) in the BTS group and 26 (53.1 %) in the ES group, p = 0.007], had a higher total in-hospital cost (78.0 ± 30.3 thousand yuan in the BTS group and 62.2 ± 46.1 thousand yuan in the ES group, p = 0.049) and more often had perineural invasion [25 (51.0%) in the BTS group and 13 (26.5%) in the ES group, p = 0.013]. Other perioperational outcomes, including surgical adverse effects (Clavien-Dindo classification) (p = 0.416), pT stage (p = 0.301), pN stage (0.639), pM stage (p = 1.000), patients who had vascular invasion [15 (30.6%) vs. 18 (36.7%), p = 0.521], patients who received adjuvant chemotherapy [26 (53.1%) vs. 25 (51.0 %), p = 0.686], total hospital stay (27.2 days vs. 24.1, p = 0.321) and 30 day mortality [1 (2.0%) vs. 1 (2.0 %), p = 1.000], did not differ between treatment groups. The eight patients with pM1 disease in the BTS group included four patients with peritoneal metastasis, two with lung metastasis, one with liver metastasis and one with both liver and lung metastasis. The eight patients with pM1 disease in the ES group after matching incuded four patients with peritoneal metastases, two with lung metastasis, one with liver metastasis and one with brain metastasis.

**Table 2:** Clinicopathological characteristics

	Before propensity score matching			After propensity score matching (1:1)		
	BTS (n = 49)	ES (n = 118)	*P*	BTS (n = 49)	ES (n = 49)	*P*
Surgical approach			0.000			0.000
Laparotomy	18 (36.7 %)	79 (66.9 %)		18 (36.7 %)	41 (83.7 %)	
Laparoscopy	31 (63.3 %)	39 (33.1 %)		31 (63.3 %)	8 (16.3 %)	
Primary resection type			0.068			0.007
Without stoma	36 (73.5 %)	69 (58.5 %)		36 (73.5 %)	23 (46.9 %)	
With stoma	13 (26.5 %)	49 (41.5 %)		13 (26.5 %)	26 (53.1 %)	
Stent procedure						
Technique failure	5 (10.2 %)			5 (10.2 %)		
Stent–related perforations	2 (4.1 %)			2 (4.1 %)		
Clinical success	42 (85.7 %)			42 (85.7 %)		
Surgical AEs (Clavien–Dindo classification)			0.447			0.416
1	0	0		0	0	
2	2 (4.1 %)	11 (9.3 %)		2 (4.1 %)	8 (16.3 %)	
3	8 (16.3 %)	3 (2.5 %)		8 (16.3 %)	0	
4	0	2 (1.7 %)		0	1 (2.0 %)	
5	1 (2.0 %)	5 (4.2 %)		1 (2.0 %)	0	
pT stage			0.136			0.301
T1	1 (2.0 %)	2 (1.7 %)		1(2.0 %)	2 (4.1 %)	
T2	1 (2.0 %)	2 (1.7 %)		1 (2.0 %)	1 (2.0 %)	
T3	19 (38.8 %)	52 (44.1 %)		19 (38.8 %)	23 (46.9 %)	
T4	28 (57.1 %)	62 (52.5 %)		28 (57.1 %)	23 (46.9 %)	
pN stage			0.655			0.639
N0	25 (51.0 %)	69 (58.5 %)		25 (51.0 %)	25 (51.0 %)	
N1	19 (38.8 %)	26 (22.0 %)		19 (38.8 %)	14 (28.6 %)	
N2	5 (10.2 %)	23 (19.5 %)		5 (10.2 %)	10 (20.4 %)	
pM stage			0.898			1.000
M0	41 (83.7 %)	98 (83.1 %)		41 (83.7 %)	41 (83.7 %)	
M1	8 (16.3 %)	20 (16.9 %)		8 (16.3 %)	8 (16.3 %)	
Vascular invasion	15 (30.6 %)	50 (42.4 %)	0.681	15 (30.6 %)	18 (36.7 %)	0.521
Perineural invasion	25 (51 %)	40 (33.9 %)	0.039	25 (51.0 %)	13 (26.5 %)	0.013
Adjuvant therapy	26 (53.1 %)	56 (47.5 %)	0.510	26 (53.1 %)	25 (51.0 %)	0.686
Total hospital stay (mean(SD), d)	27.2 (15.9)	24.7 (14.5)	0.353	27.2 (15.9)	24.1 (13.8)	0.321
Total in–hospital cost(mean(SD), 10^3^ RMB yuan)	78.0 (30.3)	61.2 (40.7)	0.004	78.0 (30.3)	62.2 (46.1)	0.049
30 day mortality	1 (2.0 %)	3 (2.5 %)	0.847	1 (2.0 %)	1 (2.0 %)	1.000

### Long‐term outcomes

**Fig. 2:**
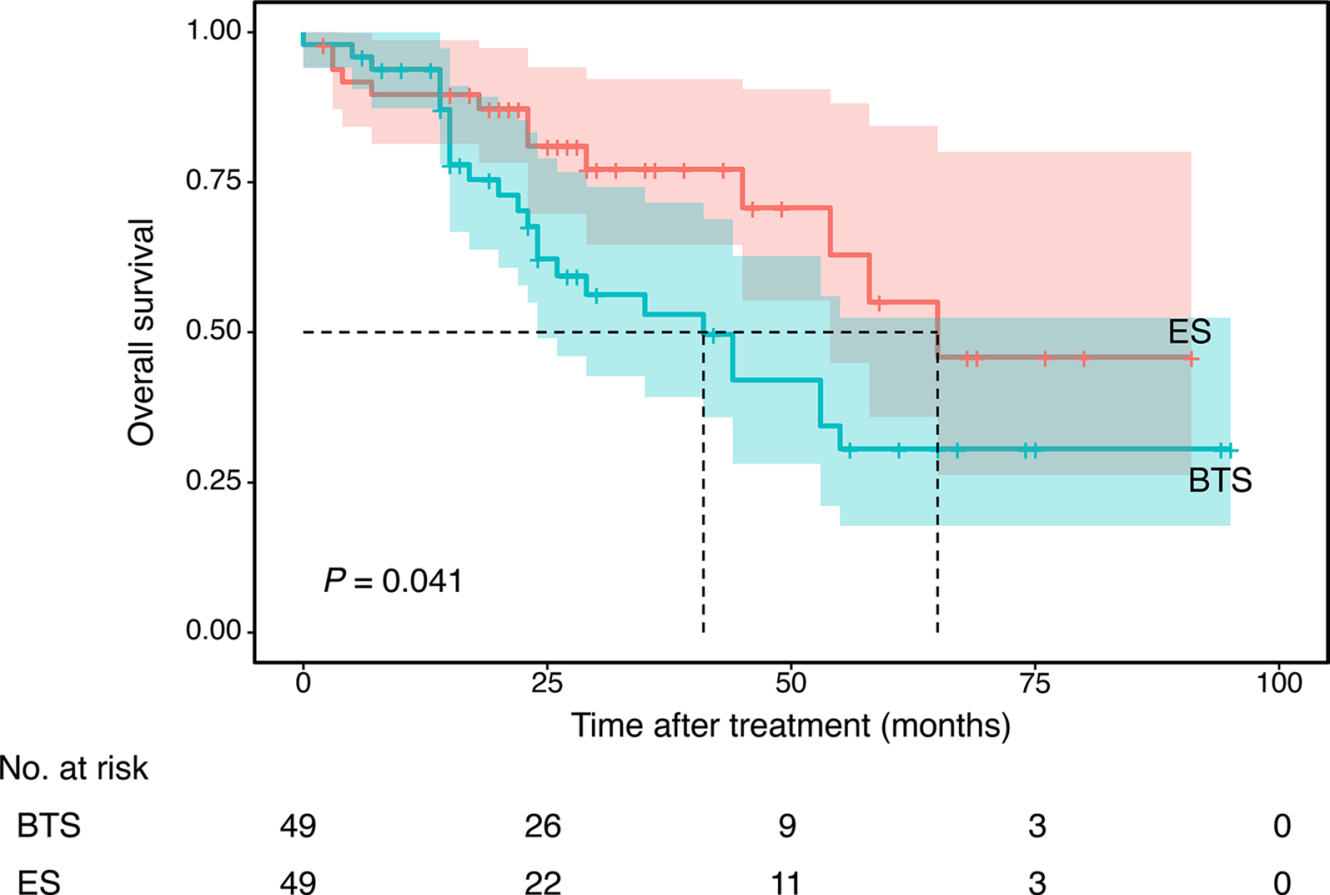
Survival curves for bridge to surgery (BTS) vs. emergency surgery (ES) (propensity score-matched patients)

The mean follow-up period was 31.7 ± 3.06 months in the BTS group and 31.5 ± 3.32 months in the ES group (p = 0.970) (Tables 3 and 4). In the PSM-matched population, overall survival was significantly less favorable for patients with stent insertion as indicated by 3-year OS (53.0 % in the BTS group and 77.2 % in the ES group, p = 0.039) and 5-year OS (30.6 % in the BTS group and 55.0 % in the ES group, p = 0.025). The median overall survival was 41 months in the BTS group and 65 months in the ES group(p = 0.041) (Fig. 2). The total recurrence rate was significantly increased in the BTS group [25 (51.0 %) vs. 13 (26.5 %), p = 0.013]. However, no significant differences were noted between the two groups in terms of regional recurrence [10 (20.4 %) vs. 4 (8.2 %), p = 0.083] and distant metastasis [15 (30.6 %) vs. 9 (18.4 %), p = 0.159].

**Table 3:** Long-term outcomes in PSM-matched patients

	BTS (n = 49)	ES (n = 49)	*P*
Mean follow–up period (mo)	31.7 ± 3.06	31.5 ± 3.32	0.970
Overall survival (%)			
At 1y	93.8 ± 3.4	89.6 ± 4.4	0.450
At 3y	53.0 ± 8.1	77.2 ± 7.0	0.039
At 5y	30.6 ± 8.4	55.0 ± 12.0	0.025
Recurrence	25 (51.0 %)	13 (26.5 %)	0.013
Regional recurrence	10 (20.4 %)	4 (8.2 %)	0.083
Distant metastasis	15 (30.6 %)	9 (18.4 %)	0.159

**Table 4:** Multivariable analysis of known risk factors for overall survival

	Hazard ratio	*P*
BTS and ES patients (After PSM analysis)		
ASA score	1.271 (0.827–1.953)	0.274
Stenting	2.309 (1.052–5.066)	0.037
Stoma creation	1.61 (0.764–3.391)	0.210
Surgical AEs	1.394 (1.053–1.845)	0.020
pTNM stage	1.706 (1.116–2.607)	0.014
Perineural invasion	0.82 (0.371–1.809)	0.622
BTS patients only		
Time from stenting to resection	1.003 (0.997,1.01)	0.315
Surgical AEs	1.678 (1.126,2.501)	0.011
pTNM stage	2.125 (1.177,3.836)	0.012
Perineural invasion	1.158 (0.42,3.195)	0.776
Adjuvant therapy	1.194 (0.447,3.191)	0.723

In multivariate Cox regression analysis, stenting (hazard ratio (HR) = 2.309 (1.052–5.066), p = 0.037), surgical AEs [HR = 1.394 (1.053–1.845), p = 0.020] and pTNM stage [HR = 1.706 (1.116–2.607), p = 0.014] were positively correlated with overall survival in matched patients. Other factors, including ASA score, stoma creation and perineural invasion, showed significant differences in univariate analysis, but no significant differences in multivariate analysis. In the BTS group only, surgical AEs (p = 0.011) and pTNM stage (p = 0.012) were associated with the overall survival estimate. Other factors that are known for predictors of postoperative survival, including age, time from stenting to resection, perineural invasion and adjuvant therapy, were also included in the analysis but showed no significance.

## Discussion

This study is a multicenter trial that analyzed retrospective data from three hospitals with considerable follow-up periods where both BTS and ES were available. The results of this observational study suggest that SEMS placement as BTS in patients with AMLCO was associated with fewer primary stoma creations, higher total in-hospital costs and more perineural invasion. Patients in the BTS group had a higher recurrence rate and poorer 3-year and 5-year overall survival, which were closely correlated with surgical adverse effects and pTNM stage.

Successful placement of the stent relies on the severity of colonic obstruction and expertise of the endoscopist. The technical (89.8 %) and clinical success (85.7 %) rates in this study were similar to those in previous studies (84.2–100 % and 78.9–100 %, respectively) [18]. Established short-term advantages of bridged surgery include less temporary and permanent stoma creation as analyzed in many meta-analyses [18, 19]. Our study confirmed that BTS significantly increased the use of laparoscopy and decreased stoma creation.

However, recent studies have failed to show beneficial effects of stenting as BTS over emergency surgery, due to uncertainty of its impact on long-term oncological outcomes [20, 21]. This originates from concerns about tumor manipulation during stent insertion, guidewire perforations during stent placement [22], stent deployment force and eventual microperforations at the proximal and distal ends of the stent [23], which may induce tumor cell dissemination locally but also in the bloodstream [13]. In our study, we found that patients with BTS had a higher risk of perineural invasion, similar to Kim’s findings [24]. In a multivariate analysis by Leibig et al. [25], perineural invasion was thought to be an independent prognostic factor of oncological outcomes in colorectal cancer. In our analysis, perineural invasion was significantly associated with the overall survival of propensity score-matched patients by univariate survival analysis (data not shown). However, the correlation was not significant in multivariate analysis. Long-term large-scale studies are needed to better investigate the correlation of perineural invasion and oncological outcomes. Stent insertion was associated with more total recurrence in this study, although the difference was not significant in regional or distant recurrence alone. Similar to the findings in our study, a recent meta-analysis [26] of 7 randomized controlled trials demonstrated that BTS significantly increased the risk of recurrence, especially distant recurrence.

To date, very few studies have reported on long-term survival after SEMS placement as a BTS due to a scarcity of clinical data and the lack of comparable studies. Femke et al. [19] found that SEMS placement as BTS did not influence 3 and 5 year overall survival in a meta-analysis. This finding is similar to Sun’s findings [12], which suggest colonic stenting did not affect 5 and 10 year survival; however, the study population is relatively small and an accurate conclusion cannot be drawn. However, in Kim’s study [27], SEMS placement negatively affected 5 year overall survival and disease-free survival (DFS) in stage II and III CRCs (5-year OS: 44 % after SEMS versus 87 % after elective surgery for nonobstructing CRC). Sabbagh et al. [21] also found that 5 year overall survival was significantly lower in the BTS group, whereas 5 year cancer-specific mortality was significantly higher (48 vs. 21 %, p = 0.02). However, no significant differences in terms of 5 year DFS were noted. In our study, we found that 3 and 5 year overall survival were significantly lower in patients who underwent SEMS as BTS than in those who underwent ES.

Colonic stent insertion also affects patient survival in multiple aspects. Avlund et al. performed a 10-year follow-up study and concluded an association between SEMS-related perforations and decreased survival [28]. The interval from SEMS to resection surgery was thought to delay the surgery and increase the rate of recurrence and survival in the study by Broholm [20]; however, further larger studies are needed to confirm the results. Postoperative adverse effects, especially infectious complications, were associated with poorer survival in patients after colorectal cancer resection [29]. SEMS insertion was also reported to be associated with increased perineural invasion [30], which is a known prognostic factor in CRCs and correlates with the findings in our study. Many clinical factors could influence the prognosis of obstructing CRCs and overall survival. An analysis by Rodrigues et al. suggests that pTMN stage IV, number of lymph nodes harvested, adjuvant therapy and surgery-related complications could influence overall survival [2]. In our study, stenting along with surgical adverse effects and pTNM stage were associated with overall survival by multivariate analysis of propensity score-matched patients.

The present study had several limitations. First, its retrospective nature may introduce selection bias and affect the results. Second, because the population after propensity score matching was relatively small, the analysis of some variables showed a wide range. The effect of SEMSs as BTSs should be cautiously interpreted. Third, although the medical records were carefully reviewed and follow-up studies were thoroughly performed the causes of death were difficult to confirm in some cases, and disease-free survival was lacking. The strengths of our studies are the homogeneity between groups, due to the use of PSM analysis and the long follow-up period.

## Conclusions

SEMS placement was associated with high technical and clinical success, similar to ES, as demonstrated by the higher primary anastomosis rate and lower stoma rates, with its possible positive effects on quality of life. However, SEMS placement as a BTS leads to more perineural invasion, higher recurrence rate and worse long-term overall survival compared to ES. These results suggest that SEMS placement should not be routinely performed in patients with potentially cured AMLCOs.

## Data Availability

The datasets used and analyzed during the current study are available from the corresponding author on reasonable request.

## Abbreviations

BTS: Bridge to surgery
SEMS: Self-expanding metal stent
ES: Emergency surgery
AMLCO: Acute malignant left-sided colonic obstruction
PSM: Propensity score matching
AE: Adverse effect
CRC: Colorectal cancer
OS: Overall survival
RCT: Randomized controlled trial
ASA: American Society of Anesthesiology
DFS: Disease-free survival
